# Zip-Code Level Disadvantage as a Predictor of Metastatic Breast Cancer at Diagnosis and Delayed Treatment Initiation

**DOI:** 10.1245/s10434-025-18693-9

**Published:** 2025-12-01

**Authors:** Priyanka Parmar, Jessica Lin, Fardeen Bhimani, Laura Jao, Michelle Sheckley, Andreina Giron, Yu Chen, Rajika Jindani, David Entenberg, Maja Oktay, Ethan Ravetch, Anjuli Gupta, Jessica Pastoriza, Maureen McEvoy, Sheldon Feldman

**Affiliations:** 1 Department of Surgery, Montefiore Medical Center, Bronx, NY USA; 2 Division of Breast Surgical Oncology, Department of Surgery, Montefiore Medical Center, Montefiore Einstein Center for Cancer Care, Bronx, NY USA; 3 Department of Pathology, Albert Einstein College of Medicine, Bronx, NY USA

## Abstract

**Background:**

Metastatic breast cancer remains a significant public health issue, associated with worse outcomes and limited treatment options. While tumor biology influences disease progression, social and geographic disparities also contribute to late-stage diagnosis. The Distressed Communities Index (DCI), a zip-code level measure of economic hardship, captures structural disadvantage more comprehensively than traditional socioeconomic indicators. This study evaluates whether higher DCI scores are associated with metastatic stage and treatment delays, independent of clinical and demographic factors.

**Patients and Methods:**

We conducted a retrospective cohort study of women aged ≥ 18 years diagnosed or treated for in situ (DCIS) and invasive breast cancer (2018–2022) at a comprehensive cancer center. Zip codes were linked to DCI scores. Multinomial logistic regression, adjusted for age, race, ethnicity, insurance status, tumor subtype, and clinical palpable mass, assessed the association between DCI and cancer stage. Analysis of covariance compared time with treatment across DCI groups.

**Results:**

Among 2024 women (38% Black, 39% Hispanic), 76.8% resided in high DCI areas. High DCI was associated with twice the likelihood of metastatic disease at diagnosis compared with localized stage (ref: low DCI, aOR 2.16, 95% CI 2.00–2.34, *p* < 0.001) after adjustment. High DCI patients also experienced longer time to treatment initiation, including adjuvant chemotherapy (119 versus 137 days, *p* = 0.024), radiation (103 versus 133 days, *p* < 0.001), and surgery (71.2 versus 87.4 days, *p* < 0.001), after adjustment for stage.

**Conclusions:**

Leveraging DCI may help identify high-risk zip codes and guide targeted screening to reduce disparities in outcomes.

Late-stage breast cancer diagnosis remains a serious public health concern, associated with worse survival outcomes and fewer treatment options.^1^ Despite improvements in early detection and treatment, a substantial number of patients continue to present with advanced disease,^2^ pointing to ongoing gaps in healthcare access and equity.

While tumor characteristics such as subtype and grade influence stage at presentation, social and geographic factors also play a critical role.^3–5^ Social determinants of health, including income, insurance status, education, and language or cultural barriers, can delay diagnosis and limit access to timely care.^4,6–9^ These challenges are particularly pronounced in underserved communities, where multiple barriers often arise throughout the cancer care continuum.^4,9^ The Distressed Communities Index (DCI) informs on this issue by providing a zip-code level composite score of economic distress.^10^ Incorporating metrics such as poverty, unemployment, housing vacancy, and educational attainment, the DCI offers a detailed view of place-based inequities. It may be especially valuable in urban areas, where census-tract-level measures have limitations, often being disproportionately influenced by a single factor such as median home value and lacking standardized weighting.^11,12^

Here we conducted a retrospective study in a predominately urban cohort to examine whether higher DCI scores are associated with more advanced breast cancer (metastatic stage) at diagnosis and with treatment delays, independent of clinical, demographic, and tumor-specific factors. By identifying geographic areas where patients are most vulnerable to poor outcomes, we aim to inform targeted interventions that promote earlier detection and timely, equitable access to care.

## Patients and Methods

### Study Population and Study Design

This study retrospectively analyzed female patients who were ≥ 18 years and diagnosed with either Tis (DCIS) or invasive breast cancer from January 2018 to December 2022 at Montefiore Medical Center/Albert Einstein College of Medicine. The study was approved by the Institutional Review Board.

### Inclusion and Exclusion Criteria

**Fig. 1:**
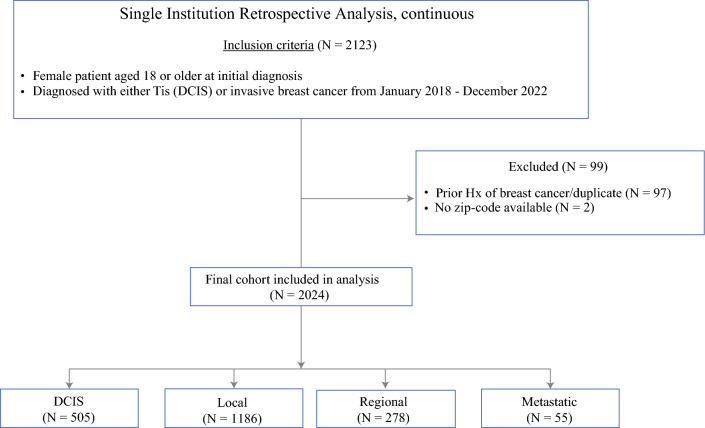
Flow diagram of study population

Patients were eligible for inclusion if they had a first-time diagnosis of either DCIS (Tis) or invasive breast cancer, as confirmed by final surgical pathology. Tumor registry sequency numbers ensured inclusion of first primary breast cancers only. The exclusion criteria included male patients, prior history of breast cancer, and patients with missing zip codes or staging information. Figure 1 shows the flow diagram of study population.

### Data Collection and Variables

Demographic, clinical, and pathologic data were obtained from the institutional tumor registry and electronic medical records. Distressed Communities Index (DCI), assigned on the basis of patient residential zip code, was used to obtain both the continuous and quintiles and were obtained from the Economic Innovation Group 2018–2022 DCI database.^10^ The DCI score incorporates metrics such as unemployment, education level, poverty rate, median income, business growth, and housing vacancies to generate a composite score ranging from 0 to 100.^10^ These data are sourced from the American Community Survey 5-year estimates (2018–2022), and the raw values are then categorized into quintiles: 1 (prosperous), 2 (comfortable), 3 (mid-tier), 4 (at risk), and 5 (distressed). For analysis, DCI was stratified into low (quintiles 1–3) and high (quintiles 4–5) categories.^10^ Clinical covariates included age, race, ethnicity, insurance status, tumor subtype, tumor stage (Tis (DCIS), localized, regional, metastatic), and presence of a palpable mass at diagnosis. Due to small sample sizes, race was grouped as white, Black, or other, with “other” including unknown, multiracial, and underrepresented racial groups. Time of diagnosis was first pathology result confirming DCIS or cancer.

### Statistical Analysis

All statistical analyses were conducted using RStudio software, version 2024.04.2+764 (Posit Software, PBC). Continuous variables were summarized as mean (SD) for normally distributed data. Group comparisons for continuous variables were performed using an unpaired two-tailed* t*-test or one-way analysis of variance (ANOVA). Categorical variables were expressed as frequencies and percentages and were analyzed using the Fisher’s exact test or chi-squared test. Statistical significance was set at a two-sided *p* < 0.05.

A multinomial logistic regression was performed to assess the association between DCI and cancer stage at diagnosis, adjusting for age, race, ethnicity, insurance status, tumor subtype, and presence of a palpable mass at diagnosis. Two models were employed: (1) model A used ductal carcinoma in situ (DCIS; Tis) as the reference group and compared localized, regional, and metastatic disease; (2) model B used localized disease as the reference group to compare regional and metastatic disease. Model A evaluated predictors of presentation at the earliest, pre-invasive stage, while model B assessed factors associated with more advanced disease among invasive cases. An analysis of covariance (ANCOVA) was used to evaluate differences in time to treatment between high and low DCI areas, adjusting for cancer stage to account for potential confounding effects. Time to treatment was calculated only among patients who received that specific treatment. Patients who did not undergo radiation, surgery, or chemotherapy were excluded from respective analyses.

## Results

### Sociodemographic and Clinical Characteristics of the Study Cohort

A total of 2024 female patients diagnosed with DCIS or invasive breast cancer between 2018 and 2022 were included in the study. Of these, 76.8% (*N* = 1555) resided in high DCI areas, while 23.2% (*N* = 469) were from low DCI areas (Table 1). Patients from high DCI areas were significantly more likely to be Black (43.2%) and Hispanic (45.2%), whereas those from low DCI areas were predominantly white (64.2%) (*p* < 0.001). Insurance status also differed significantly (*p* < 0.001), with Medicare/Medicaid coverage being more common in high DCI patients (64.7% versus 49.7%). No significant differences were found in the distribution of age (*p* = 0.842), palpable masses on presentation, tumor subtypes (*p* = 0.288), and stage (*p* = 0.626) across DCI groups (Table 1).

**Table 1:** Clinicodemographic variables of entire cohort, stratified by DCI (low versus high)

	Low DCI	High DCI	Overall	*P* value
	(*N* = 469)	(*N *= 1555)	(*N* = 2024)	
*Year of diagnosis*				
2018	116 (24.7%)	389 (25.0%)	505 (25.0%)	0.81
2019	107 (22.8%)	301 (19.4%)	408 (20.2%)	
2020	75 (16.0%)	239 (15.4%)	314 (15.5%)	
2021	75 (16.0%)	301 (19.4%)	376 (18.6%)	
2022	96 (20.5%)	325 (20.9%)	421 (20.8%)	
*Age at diagnosis*				
< 45	35 (7.5%)	140 (9.0%)	175 (8.6%)	0.84
45–54	106 (22.6%)	307 (19.7%)	413 (20.4%)	
55–64	135 (28.8%)	472 (30.4%)	607 (30.0%)	
65+	193 (41.2%)	636 (40.9%)	829 (41.0%)	
*Race*				
White	301 (64.2%)	733 (47.1%)	1034 (51.1%)	< 0.001
Black	98 (20.9%)	672 (43.2%)	770 (38.0%)	
Other	70 (14.9%)	150 (9.6%)	220 (10.9%)	
*Ethnicity*				
Non-Hispanic	361 (77.0%)	823 (52.9%)	1184 (58.5%)	< 0.001
Hispanic	92 (19.6%)	703 (45.2%)	795 (39.3%)	
Unknown	16 (3.4%)	29 (1.9%)	45 (2.2%)	
*Insurance status*				
Private	230 (49.0%)	519 (33.4%)	749 (37.0%)	< 0.001
Medicare/Medicaid	233 (49.7%)	1006 (64.7%)	1239 (61.2%)	
Not Insured/Other	6 (1.3%)	30 (1.9%)	36 (1.8%)	
*Palpable mass on presentation*				
No	314 (67.0%)	1037 (66.7%)	1351 (66.7%)	0.86
Yes	132 (28.1%)	459 (29.5%)	591 (29.2%)	
Unknown	23 (4.9%)	59 (3.8%)	82 (4.1%)	
*Subtype*				
HR+/HER2-	298 (63.5%)	911 (58.6%)	1209 (59.7%)	0.25
HR+/HER2 unknown	93 (19.8%)	354 (22.8%)	447 (22.1%)	
HER2+	36 (7.7%)	100 (6.4%)	136 (6.7%)	
TNBC	37 (7.9%)	182 (11.7%)	219 (10.8%)	
Unknown	5 (1.1%)	8 (0.5%)	13 (0.6%)	
*Stage at diagnosis*				
DCIS	111 (23.7%)	394 (25.3%)	505 (25.0%)	0.63
Local	286 (61.0%)	900 (57.9%)	1186 (58.6%)	
Regional	65 (13.9%)	213 (13.7%)	278 (13.7%)	
Metastatic	7 (1.5%)	48 (3.1%)	55 (2.7%)	

### Stage at Diagnosis by Subtype and Clinicopathological and Socioeconomic Measures

When stratified by cancer stage at diagnosis (Table 2), significant differences were observed in demographic and clinical characteristics. Race was significantly associated with stage at diagnosis (*p* < 0.001, chi-squared test). Among patients with DCIS, 43.4% were white and 44.4% were Black; for localized disease, 54.7% were white and 35.2% were Black; for regional disease, 50.0% were white and 36.3% were Black; and among those with metastatic disease, 49.1% were white and 49.1% were Black.

**Table 2:** Clinicodemographic variables of entire cohort, stratified by stage at diagnosis

	DCIS	Local	Regional	Metastatic	Overall	*P* value
	(*N *= 505)	(*N *= 1186)	(*N *= 278)	(*N *= 55)	(*N *= 2024)	
*Year of diagnosis*						
2018	129 (25.5%)	311 (26.2%)	55 (19.8%)	10 (18.2%)	505 (25.0%)	0.13
2019	112 (22.2%)	236 (19.9%)	52 (18.7%)	8 (14.5%)	408 (20.2%)	
2020	58 (11.5%)	194 (16.4%)	48 (17.3%)	14 (25.5%)	314 (15.5%)	
2021	100 (19.8%)	207 (17.5%)	55 (19.8%)	14 (25.5%)	376 (18.6%)	
2022	106 (21.0%)	238 (20.1%)	68 (24.5%)	9 (16.4%)	421 (20.8%)	
*Age at diagnosis*						
< 45	37 (7.3%)	93 (7.8%)	39 (14.0%)	6 (10.9%)	175 (8.6%)	0.047
45–54	99 (19.6%)	232 (19.6%)	69 (24.8%)	13 (23.6%)	413 (20.4%)	
55–64	159 (31.5%)	367 (30.9%)	69 (24.8%)	12 (21.8%)	607 (30.0%)	
65+	210 (41.6%)	494 (41.7%)	101 (36.3%)	24 (43.6%)	829 (41.0%)	
*Race*						
White	219 (43.4%)	649 (54.7%)	139 (50.0%)	27 (49.1%)	1034 (51.1%)	< 0.001
Black	224 (44.4%)	418 (35.2%)	101 (36.3%)	27 (49.1%)	770 (38.0%)	
Other	62 (12.3%)	119 (10.0%)	38 (13.7%)	1 (1.8%)	220 (10.9%)	
*Ethnicity*						
Non-hispanic	308 (61.0%)	697 (58.8%)	146 (52.5%)	33 (60.0%)	1184 (58.5%)	< 0.001
Hispanic	176 (34.9%)	477 (40.2%)	120 (43.2%)	22 (40.0%)	795 (39.3%)	
Unknown	21 (4.2%)	12 (1.0%)	12 (4.3%)	0 (0%)	45 (2.2%)	
*Insurance status*						
Private	221 (43.8%)	423 (35.7%)	88 (31.7%)	17 (30.9%)	749 (37.0%)	0.003
Medicare/medicaid	270 (53.5%)	746 (62.9%)	187 (67.3%)	36 (65.5%)	1239 (61.2%)	
Not insured/other	14 (2.8%)	17 (1.4%)	3 (1.1%)	2 (3.6%)	36 (1.8%)	
*Palpable mass on presentation*						
No	393 (77.8%)	788 (66.4%)	147 (52.9%)	23 (41.8%)	1351 (66.7%)	< 0.001
Yes	30 (5.9%)	398 (33.6%)	131 (47.1%)	32 (58.2%)	591 (29.2%)	
Unknown	82 (16.2%)	0 (0%)	0 (0%)	0 (0%)	82 (4.1%)	
*Subtype*						
HR+/HER2-	28 (5.5%)	929 (78.3%)	221 (79.5%)	31 (56.4%)	1209 (59.7%)	< 0.001
HR+/HER2 unknown	430 (85.1%)	14 (1.2%)	2 (0.7%)	1 (1.8%)	447 (22.1%)	
HER2+	2 (0.4%)	103 (8.7%)	26 (9.4%)	5 (9.1%)	136 (6.7%)	
TNBC	35 (6.9%)	138 (11.6%)	28 (10.1%)	18 (32.7%)	219 (10.8%)	
Unknown	10 (2.0%)	2 (0.2%)	1 (0.4%)	0 (0%)	13 (0.6%)	
*DCI score (quintile)*						
Low DCI (1-3)	111 (22.0%)	286 (24.1%)	65 (23.4%)	7 (12.7%)	469 (23.2%)	0.36
High DCI (4-5)	394 (78.0%)	900 (75.9%)	213 (76.6%)	48 (87.3%)	1555 (76.8%)	
*DCI score (continuous)*						
Mean (SD)	68.5 (19.0)	66.9 (20.2)	69.5 (18.2)	71.6 (16.0)	67.8 (19.6)	0.32
Median [Min, Max]	76.5 [4.60, 93.7]	73.3 [2.80, 93.7]	77.1 [4.60, 92.5]	73.3 [18.0, 92.4]	74.9 [2.80, 93.7]	

Insurance status was also significantly associated with stage at diagnosis (*p* = 0.005, chi-squared test). Patients with Medicare/Medicaid accounted for a larger proportion of those diagnosed with regional (67.3%) and metastatic disease (65.5%) compared with DCIS (53.5%). Palpable mass at presentation was significantly associated with more advanced disease (*p* < 0.001, chi-squared test), increasing from 5.9% in DCIS to 33.6% in localized cancer and 58.2% in metastatic disease.

Tumor subtype distribution differed significantly by stage at diagnosis (*p* < 0.001, chi-squared test). HR+/HER2− was the most common subtype across all stages but was more frequently observed in localized (78.3%) and regional disease (79.5%), whereas TNBC was significantly overrepresented in metastatic cases (32.7%). HER2+ tumors accounted for 6.2% of metastatic cases, compared with 1.9% in regional disease and 1.2% in localized disease.

DCI quintile analysis showed no statistically significant association between DCI quintile and stage at diagnosis (*p* = 0.36, chi-squared test). However, a trend was observed where 87.3% of patients diagnosed with metastatic breast cancer resided in high DCI areas, compared with 76.9% in regional disease, 75.9% in localized disease, and 78.0% in DCIS. When assessed as a continuous variable, mean DCI scores increased with stage progression, but this association was not statistically significant (*p* = 0.32, *t*-test). Mean DCI scores were 68.5 (± 19.0) for DCIS, 66.9 (± 20.2) for localized disease, 69.5 (± 18.2) for regional disease, and 71.6 (± 18.2) for metastatic stage (Table 3).

**Table 3:** Multinomial regression analysis with predictors of advanced stage: model A compares predictors of invasive breast cancer stages (local, regional, and metastatic) relative to ductal carcinoma in situ (Tis–DCIS); model B compares predictors of more advanced disease (regional and metastatic) relative to local stage breast cancer

	Model A (ref: DCIS)						Model B (ref: local stage)			
	Local	*P* value	Regional	*P* value	Metastatic	*P* value	Regional	*P* value	Metastatic	*P* value
Year of diagnosis (continuous)	0.94 (0.94, 0.94)	*p* < 0.001	1.08 (1.08, 1.08)	*p* < 0.001	1.02 (1.02, 1.02)	*p* < 0.001	1.12 (1.12, 1.12)	*p* < 0.001	1.02 (1.02, 1.02)	*p* < 0.001
Age > 50 (ref: < 50)	1.09 (0.91, 1.32)	*p *= 0.35	0.59 (0.52, 0.68)	*p* < 0.001	1.06 (1.04, 1.09)	*p* < 0.001	0.59 (0.43, 0.80)	*p* < 0.001	1.00 (0.94, 1.06)	*p* = 0.95
Black Race (ref: White)	0.48 (0.40, 0.58)	*p* < 0.001	0.69 (0.56, 0.85)	*p* < 0.001	0.55 (0.52, 0.59)	*p* < 0.001	1.34 (0.96, 1.88)	*p *= 0.09	1.06 (0.76, 1.48)	*p *= 0.71
Other Race (ref: White)	0.76 (0.66, 0.87)	*p* < 0.001	1.09 (0.99, 1.22)	*p* = 0.09	0.16 (0.16, 0.16)	*p* < 0.001	1.41 (0.87, 2.28)	*p* = 0.16	0.20 (0.20, 0.21)	*p* < 0.001
Hispanic (ref: non-Hispanic)	0.87 (0.72, 1.05)	*p *= 0.15	1.22 (1.00, 1.48)	*p* = 0.05	0.70 (0.66, 0.74)	*p *< 0.001	1.31 (0.95, 1.81)	*p *= 0.10	0.88 (0.64, 1.22)	*p *= 0.44
Medicare/Medicaid (ref: private)	1.21 (0.99, 1.48)	*p *= 0.07	1.59 (1.24, 2.04)	*p* < 0.001	1.24 (1.17, 1.32)	*p *< 0.001	1.27 (0.94, 1.70)	*p *= 0.12	0.99 (0.67, 1.47)	*p* = 0.97
Uninsured (ref: private)	0.55 (0.54, 0.55)	*p* < 0.001	0.69 (0.68, 0.69)	*p *< 0.001	1.96 (1.94, 1.97)	*p *< 0.001	1.05 (1.03, 1.07)	*p *< 0.001	2.82 (2.70, 2.94)	*p *< 0.001
Palpable Mass (ref: non palpable)	6.64 (5.69, 7.75)	*p *< 0.001	11.44 (10.14, 12.90)	*p* < 0.001	17.75 (17.46, 18.05)	*p *< 0.001	1.67 (1.27, 2.20)	*p* < 0.001	2.54 (2.14, 3.02)	*p *< 0.001
TNBC subtype (ref: HR+)*	2.36 (1.85, 3.01)	*p *< 0.001	1.93 (1.68, 2.21)	*p* < 0.001	8.36 (7.97, 8.77)	*p *< 0.001	0.81 (0.52, 1.26)	*p *= 0.35	3.51 (2.57, 4.78)	*p* < 0.001
HER2+ subtype (ref: HR+/HER2-)	–	–	–	–	–	–	1.05 (0.66, 1.67)	*p* = 0.84	1.39 (1.31, 1.47)	*p *< 0.001
High DCI (ref: low)	0.97 (0.79, 1.18)	*p *= 0.73	0.85 (0.72, 0.99)	*p *= 0.04	1.98 (1.94, 2.02)	*p* < 0.001	0.88 (0.62, 1.25)	*p* = 0.48	2.16 (2.00, 2.34)	*p *< 0.001

Values are represented as aOR (95% CI) where aOR is adjusted odds ratio and 95% CI is 95% confidence intervals

*In model A the reference is HR+ given that many patients in DCIS group lacked HER2 status, in model B the reference is HR+/HER2-

### High DCI is Associated with Metastatic Stage Breast Cancer at Initial Diagnosis, Compared with DCIS or Local Stage

Patients residing in high DCI zip codes had higher odds of presenting with metastatic stage compared with both DCIS (aOR 1.98, 95% CI 1.94–2.02, *p* < 0.001, model A) and localized stage (aOR 2.16, 95% CI 2.00–2.34, *p* < 0.001, model B). However, high DCI was inversely associated with regional stage compared with DCIS (aOR 0.85, 95% CI 0.72–0.99, *p* = 0.04, model A) but was not associated with regional versus localized stage (*p* = 0.48, model B).

Older age (≥ 50 years) was associated with metastatic stage compared with DCIS (aOR 1.06, 95% CI 1.04–1.09, *p* < 0.001, model A) but not metastatic stage compared with localized stage (*p* = 0.95, model B). Black race and Hispanic ethnicity were not associated with metastatic versus localized stage (Black: *p* = 0.71; Hispanic: *p* = 0.44; model B) or regional versus localized stage (Black: *p* = 0.09, Hispanic: *p* = 0.10, model B), but were associated with lower odds of metastatic stage compared with DCIS (Black: aOR 0.55, 95% CI 0.52–0.59, *p* < 0.001; Hispanic: aOR 0.70, 95% CI 0.66–0.74, *p* < 0.001, model A) and similarly with regional stage versus DCIS (Black: aOR 0.69, 95% CI 0.56–0.85, *p* < 0.001; Hispanic: aOR 0.80, 95% CI 0.69–0.93, *p* = 0.004, model A).

Medicare/Medicaid coverage was associated with metastatic stage compared with DCIS (aOR 1.24, 95% CI 1.17–1.32, *p* < 0.001, model A) but not metastatic compared with localized stage (*p* = 0.97, model B). Lack of insurance predicted higher odds of metastatic presentation compared with both DCIS (aOR 1.96, 95% CI 1.94–1.97, *p* < 0.001, model A) and localized disease (aOR 2.82, 95% CI 2.70–2.94, *p* < 0.001, model B).

Presenting with a palpable mass was associated with higher odds across stages: localized versus DCIS (aOR 6.64, 95% CI 5.69–7.75, *p* < 0.001, model A), regional versus DCIS (aOR 11.44, 95% CI 10.14–12.90, *p* < 0.001, model A), and metastatic versus DCIS (aOR 17.75, 95% CI 17.46–18.05, *p* < 0.001, model A); and with regional (aOR 1.67, 95% CI 1.27–2.20, *p* < 0.001, model B) and metastatic (aOR 2.54, 95% CI 2.14–3.02, *p* < 0.001, model B) compared with localized disease. Similarly, TNBC subtype showed increasing odds of advanced stage: localized versus DCIS (aOR 2.36, 95% CI 1.85–3.01, *p* < 0.001), regional versus DCIS (aOR 1.93, 95% CI 1.68–2.21, *p* < 0.001, model A), metastatic versus DCIS (aOR 8.36, 95% CI 7.97–8.77, *p* < 0.001, model A), and metastatic versus localized stage (aOR 3.51, 95% CI 2.57–4.78, *p* < 0.001, model B). HER2+ subtype was associated with metastatic compared with localized stage (aOR 1.39, 95% CI 1.31–1.47, *p* < 0.001, model B).

### DCI Score is Associated with Delays in Treatment Initiation, Particularly Time to Radiation and Surgery

Delays in time to treatment (if given) were assessed by comparing the time between date of diagnosis and the initiation of radiation therapy, surgery, adjuvant chemotherapy, and neoadjuvant chemotherapy for patients with invasive breast cancer. This time to treatment was analyzed between the high and low DCI groups, with adjustments for stage, as presented in Table 4.

**Table 4:** Adjusted time to treatment by high versus low DCI, adjusted for stage at diagnosis

	Adjusted Mean* (95% CI)		Difference between High–Low DCI (days)	*P* value
	Low DCI	High DCI		
Time to radiation (days)	103 (81.5–125)	133 (114.5–152)	30	< 0.001
Time to surgery (days)	71.2 (55.7–86.7)	87.4 (72.5–102.4)	16.2	< 0.001
Time to adjuvant chemotherapy (days)	119 (92.9–145)	137 (113.3–161)	18	0.024
Time to neoadjuvant chemotherapy (days)	28.2 (− 44 to 101)	75.8 (48.7–103)	47.6	0.21

^*^Adjusting for stage of diagnosis for invasive breast cancer

Patients from high DCI areas had significantly longer time to radiation (133 days, 95% CI 114.5–152) compared with those from low DCI areas (103 days, 95% CI 81.5–125), with an adjusted difference of 30 days (*p* < 0.001). Time to surgery was also significantly prolonged in high DCI patients (87.4 days, 95% CI 72.5–102.4) versus low DCI patients (71.2 days, 95% CI 55.7–86.7), showing a difference of 16.2 days (*p* < 0.001).

Time to adjuvant chemotherapy was significantly prolonged in high DCI patients (137 days, 95% CI 113.3–161) versus low DCI patients (119 days, 95% CI 92.2–145), showing a difference of 18 days (*p* = 0.024). The time to neoadjuvant chemotherapy was longer for high DCI patients (75.8 days, 95% CI 48.7–103) compared with low DCI patients (28.2 days, 95% CI −44 to 101), but this difference did not reach statistical significance (*p* = 0.21).

### DCI Score Not Associated with Overall Survival by Stage

Lastly, we sought to understand whether high DCI was associated with worse overall survival by stage, as presented in Table 5. Among patients with DCIS, survival was 100% in the low DCI group and 99% in the high DCI group (*p* = 0.62). In local stage disease, survival was similarly high: 98.6% for low DCI versus 97.9% for high DCI (*p* = 0.58). For regional stage, survival was 92.3% in the low DCI group and 95.3% in the high DCI group (*p* = 0.53). Among patients with metastatic disease, survival was lower overall, with 85.7% survival in the low DCI group compared with 70.8% in the high DCI group; however, this difference was not statistically significant (*p* = 0.66).

**Table 5:** Overall survival by high versus low DCI score, stratified by stage

	Low DCI	High DCI	*P* value
DCIS	100%	99%	0.62
Local	98.60%	97.90%	0.58
Regional	92.30%	95.30%	0.53
Metastatic	85.70%	70.80%	0.66

## Discussion

Here we show that in a predominate urban cohort, patients residing in zip codes with higher Distressed Communities Index (DCI) scores had nearly twice the odds of presenting with metastatic breast cancer compared with those with local stage and DCIS, even after adjusting for clinical and biologic factors including age, race, ethnicity, insurance status, tumor subtype, and the presence of a palpable mass. Additionally, patients from high DCI areas experienced delays in treatment initiation, including surgery and radiation, after adjusting for stage. These findings underscore the independent and important role that zip-code level social and economic distress plays in shaping breast cancer presentation and outcomes.

Given that 76.8% of patients in our cohort resided in high DCI areas, these findings likely reflect the socioeconomic profile of our hospital catchment area, which predominantly serves urban communities with elevated levels of social and economic distress. This context may also contribute to the relatively smaller proportion of patients from low DCI areas in our study. Our findings align with prior research linking structural and geographic disadvantage to late-stage cancer diagnoses.^13–18^ However, results across the literature have varied depending on the geographic unit of analysis, such as county, census tract, or zip code, urban or rural setting,^19–21^ and the specific variables and weighting methods used to construct these deprivation indices.^10,22–24^ Among the most widely used tools are the Area Deprivation Index (ADI) and the Social Vulnerability Index (SVI). ADI has been associated with late-stage breast and colorectal cancer diagnoses and breast cancer disparities in Indiana and Kentucky.^13,14^ Although our findings are in line with some of these previous studies, despite these associations, census-tract-based tools have limitations, particularly in densely populated cities.^11,12^ In urban settings such as New York, census tracts often encompass both affluent and socioeconomically distressed neighborhoods. This aggregation can obscure high-risk neighborhoods and create a false sense of equity. Recent studies of the ADI have shown that, in urban areas, scores can be disproportionately influenced by a single domain, such as median home value, rather than capturing the broader spectrum of socioeconomic disadvantage.^12^ These issues are compounded by a lack of standardized weighting during score these calculations, which may reduce the accuracy of the index.^11^

In contrast, the DCI uses zip-code-level data and incorporates broader indicators of economic vitality such as job growth, business activity, housing vacancy, and educational attainment.^10^ This granularity may better reflect the lived experience of patients, especially in economically heterogeneous urban environments. Although DCI has been less commonly used in oncology research, a growing body of evidence supports its relevance to cancer care, from screening utilization to surgical outcomes and hospital readmissions.^25–30^ For example, Herbert et al. found that age-adjusted breast cancer mortality was highest in the most distressed communities, and that screening rates improved as DCI scores improved.^25^ Other studies have shown that higher DCI scores independently predict postoperative complications even after adjusting for NSQIP risk calculators,^26^ and are associated with hospital readmissions and healthcare utilization across a range of conditions.^27–30^

One likely contributor to the link between high DCI and metastatic stage breast cancer diagnosis is delayed or reduced access to screening. While we did not have patient-level screening data, prior studies have shown that irregular or delayed screening is a strong predictor of late-stage presentation.^31,32^ Zuley et al. found that late-stage breast cancer occurred in 9% of women who underwent annual screening, compared with 14% and 19% in those screened biennially or intermittently, respectively, regardless of age, race, or menopausal status.^31^ Similarly, Mobley et al. identified geographic “hot spots” for late-stage presentation in areas with limited access to mammography and a high proportion of lower income, Black, Hispanic, or Native American residents.^32^ Herbert et al. also found that as DCI scores improved, so did breast cancer screening rates.^25^ These findings, together with our own, support the hypothesis that distressed communities frequently experience overlapping barriers to early detection and therefore present with late-stage diagnosis.

Crucially, these disparities extended beyond diagnosis to treatment initiation. Even after adjusting for stage, patients from high DCI communities experienced significantly longer times to both surgery and radiation. Optimal benchmarks for treatment are < 60 days for surgery^33^ and < 120 days for chemotherapy,^34^ yet our data show an average time to adjuvant chemotherapy in high DCI patients was 137 days, reflecting almost a 3-week delay. For surgery, high DCI patients had a notable 3 week delay when compared with low DCI group. Similarly, high DCI patients were almost 1 month delayed getting radiation. These findings suggest that socioeconomic and structural factors influence not only stage at diagnosis, but also timely access to definitive treatment.^4^ Regarding overall survival, while we did not observe significant differences between low and high DCI groups by stage, prior national studies using Centers for Disease Control and Prevention (CDC) data have shown higher DCI is associated with increased age-adjusted death rates for breast and colon cancer, suggesting our analysis may have been underpowered.^25^

Interestingly, in our adjusted model, comparing DCIS with regional stage showed high DCI was associated with lower odds of regional stage compared with DCIS (aOR 0.85, 95% CI 0.72–0.99, *p* = 0.036). At first glance, this seems to contradict our finding that high DCI is associated with more advanced disease. However, using DCIS as the reference category across the full disease spectrum may mask clinically relevant differences. Indeed, when we directly compared regional versus localized invasive disease, a comparison more clinically relevant, DCI was not significantly associated with disease stage. Future research should clarify whether the higher odds of DCIS diagnoses observed in high DCI communities represent incidental screening findings or reflect an underdiagnosis of more advanced invasive cancers.

Although DCI as a composite measure was not significantly associated with stage across all comparisons, individual components that define high DCI, such as Black race, non-private insurance, and more aggressive tumor subtypes, were each independently associated with more advanced stage at diagnosis. This suggests that while DCI captures community-level disadvantage, its composite nature may dilute the influence of specific high-risk sociodemographic and biologic factors when analyzed collectively.

Our findings also point to several potential interventions to mitigate structural disparities in high DCI communities. Given the link between higher DCI and both metastatic stage diagnosis and treatment delays, targeted outreach may be critical. Mobile mammography units in high DCI zip codes could reduce barriers to screening, such as transportation and time off work, and have been shown to increase uptake in underserved populations.^35^ Additionally, integrating community health workers and patient navigators into care pathways may help reduce treatment delays.^36^ These roles can provide culturally sensitive, individualized support, assisting with appointment scheduling, transportation, insurance navigation, and addressing social needs such as housing or childcare.^36^ Future studies should evaluate the implementation and impact of such interventions to reduce inequities in both early detection and timely access to treatment.

To our knowledge, we are the first to report the association between DCI and metastatic stage breast cancer outcomes in an urban population. DCI may be a valuable tool for more precisely identifying high-risk communities. While DCI has not been as widely used in cancer disparities research, its design and zip-code level granularity offer advantages, particularly in urban and economically diverse regions. Future research should continue to evaluate the predictive utility of DCI across cancer types and geographic settings.

### Limitations

This study has several limitations. First, while DCI provides a granular view of socioeconomic distress at the zip-code level, it remains an area-based measure and does not capture individual-level socioeconomic status. Geographic proxies can overlook substantial variability in individual-level disparities.^37^ Second, zip-code level data may still obscure neighborhood-level differences, especially in dense urban areas, due to the modifiable areal unit problem (MAUP) in which geographic boundaries can influence outcomes and introduce misclassification.^38^ Third, although we adjusted for various clinical and demographic variables, we lacked data on factors such as mammography access, provider availability, transportation, and health literacy, which likely impact diagnosis and treatment timelines. Our study also overlapped with the coronavirus disease 2019 (COVID-19) pandemic (2019–2021), which disrupted care and may have disproportionately impacted disadvantaged communities. Although our findings remained significant, we cannot fully isolate the pandemic’s effect, though prior studies report exacerbated geographic disparities.^39,40^ Additionally, the number of metastatic breast cancer cases was small, limiting statistical power. Future efforts should expand beyond a single institution to include a larger metastatic cohort. Finally, our urban Bronx-based population may limit generalizability, as most patients lived in distressed zip codes. Broader studies across rural and mixed populations, with a range of DCI scores, are needed to determine whether these patterns persist nationally.

## Conclusions

The study demonstrates that higher DCI scores are significantly associated with metastatic stage breast cancer diagnoses and treatment delays. These findings underscore the importance of considering socioeconomic distress, particularly DCI scores, in healthcare delivery and highlight the need for targeted interventions to improve outcomes in distressed communities. Identifying high-risk areas through DCI can guide more equitable healthcare strategies and ultimately help reduce the burden of metastatic breast cancer.
